# ‘Mothers moving towards empowerment’ intervention to reduce stigma and improve treatment adherence in pregnant women living with HIV in Botswana: study protocol for a pragmatic clinical trial

**DOI:** 10.1186/s13063-020-04676-6

**Published:** 2020-10-07

**Authors:** Ohemaa B. Poku, Ari R. Ho-Foster, Patlo Entaile, Supriya Misra, Haitisha Mehta, Shathani Rampa, Melody Goodman, Tonya Arscott-Mills, Evan Eschliman, Valerie Jackson, Tadele Melese, Timothy D. Becker, Marlene Eisenberg, Bruce Link, Vivian Go, Philip Renison Opondo, Michael B. Blank, Lawrence H. Yang

**Affiliations:** 1 Johns Hopkins University, Baltimore, MD United States; 2 Perelman School of Medicine, University of Pennsylvania, Philadelphia, PA United States; 3 University of Botswana, Gaborone, Botswana; 4 Botswana-UPenn Partnership, Gaborone, Botswana; 5 New York University, New York, NY United States; 6 Columbia University, New York, NY United States; 7 University of California San Francisco, San Francisco, CA United States; 8 Icahn School of Medicine at Mount Sinai, New York, NY United States; 9 University of Pennsylvania, Philadelphia, PA United States; 10 University of California Riverside, Riverside, CA USA; 11 University of North Carolina at Chapel Hill, Chapel Hil, NC USA; 12 Columbia University Mailman School of Public Health, New York, NY USA

**Keywords:** HIV/AIDS, Intervention, Culture, Botswana, Postpartum, Stigma, ART

## Abstract

**Background:**

With high rates of HIV and multiple vulnerable subgroups across diverse settings, there is a need for culturally based, HIV stigma reduction interventions. Pregnant women who are living with HIV are especially in need of services to protect not only their own but also their children’s lives. Uptake of HIV services worldwide is hindered by stigma towards persons living with HIV/AIDS. While cultural context plays a key role in shaping HIV stigma, these insights have not yet been fully integrated into stigma reduction strategies. By utilizing the “What Matters Most” stigma framework, we propose that an intervention to counter culturally salient aspects of HIV stigma will improve treatment adherence and other relevant outcomes. A pragmatic clinical trial in Botswana will evaluate the “Mothers Moving towards Empowerment” (MME) intervention, which seeks to address HIV stigma in Botswana and to specifically engage pregnant mothers so as to promote antiretroviral therapy (ART) adherence in the postpartum period.

**Methods:**

This study will test MME against treatment as usual (TAU) among pregnant mothers diagnosed with HIV and their infants. Outcomes will be assessed during pregnancy and 16 weeks postpartum. Women who meet eligibility criteria are assigned to MME or TAU. Women assigned to MME are grouped with others with similar estimated delivery dates, completing up to eight intervention group sessions scheduled before week 36 of their pregnancies. Primary outcomes among mothers include (i) reducing self-stigma, which is hypothesized to mediate improvements in (ii) psychological outcomes (quality of life, depression and social functioning), and (iii) adherence to antenatal care and ART. We will also examine a set of follow-up infant birth outcomes (APGAR score, preterm delivery, mortality (at < 16 weeks), birth weight, vaccination record, and HIV status).

**Discussion:**

Our trial will evaluate MME, a culturally based HIV stigma reduction intervention using the “What Matters Most” framework, to reduce stigma and improve treatment adherence among pregnant women and their infants. This study will help inform further refinement of MME and preparation for a future large-scale, multisite, randomized controlled trial (RCT) in Botswana.

**Trial registration:**

ClinicalTrials.gov NCT03698981. Registered on October 8, 2018

## Administrative information

**Table:** 

Title	A Pragmatic Clinical Trial Evaluating the ‘Mothers Moving towards Empowerment’ Intervention to Reduce Stigma and Improve Treatment Adherence in Pregnant Women Living with HIV in Botswana: Study Protocol for a Pragmatic Clinical Trial
Trial registration	As of submission, this protocol is version 2 and is registered with www.clinicaltrials.gov (NCT03698981) as of October 8, 2018. Recruitment began in March 15, 2019 and will be completed as of October 31, 2020.
Date and version identifier	Version 2_18.11.28
Funding	This study is funded by the National Institutes of Health/ Fogarty (R21TW011084; PI: Dr. Lawrence Yang).
Author details	Ohemaa B. Poku, MPH, Johns Hopkins University, Baltimore, MD, United States Ari R. Ho-Foster, MSc, University of Botswana, Gaborone, Botswana; Perelman School of Medicine, University of Pennsylvania, Philadelphia, PA, United States Patlo Entaile, BSW, Botswana-UPenn Partnership, Gaborone, Botswana Supriya Misra, ScD, New York University, New York, NY, United States Haitisha Mehta, MA, Columbia University, New York, NY, United States Shathani Rampa, MSc, University of Botswana, Gaborone, Botswana Melody Goodman, PhD, New York University, New York, NY, United States Tonya Arscott-Mills, MD, University of Pennsylvania, United States; University of Botswana, Gaborone, Botswana Evan Eschliman, MS, Columbia University, New York, NY, United States Valerie Jackson, PhD, University of California San Francisco, San Francisco, CA, United States Tadele Melese, MD, MMed (OBGY), University of Botswana, Gaborone, Botswana Timothy D. Becker, MD, Icahn School of Medicine at Mount Sinai, New York, NY, United States Marlene Eisenberg, PhD, University of Pennsylvania, Philadelphia, PA, United States Bruce Link, PhD, University of California Riverside, Riverside, CA, United States Vivian Go, PhD, University of North Carolina at Chapel Hill, Chapel Hill, NC , United States Philip Renison Opondo, MBCHB, MMed (Psych), University of Botswana, Gaborone, Botswana Michael B. Blank, PhD, University of Pennsylvania, Philadelphia, PA, United States Lawrence H. Yang, PhD, New York University, New York, NY, United States; Columbia University Mailman School of Public Health, New York, NY, United States
Name and contact information for the trial sponsor	*National Institute of Mental Health* 6001 Executive BoulevardRockville, MD 20852 *Fogarty International Center* Division of International Training and ResearchFogarty International CenterNational Institutes of Health31 Center Drive, MSC 2220Bethesda, MD 20892 Phone: 301-496-1492
Role of study sponsor and funders, if any, in study design; collection, management, analysis, and interpretation of data; writing of the report; and the decision to submit the report for publication, including whether they will have ultimate authority over any of these activities	Not applicable.

## Introduction

With the continuing high rates of HIV and the identification of vulnerable subgroups across a diversity of settings, there is an urgent need for culturally based HIV stigma reduction interventions. Pregnant women who are living with HIV are especially in need of services in order to protect not only their own but their children’s lives. Uptake of HIV services worldwide is hindered by stigma towards persons living with HIV/AIDS (PLWHA) [1–7], and while cultural context plays a key role in shaping HIV stigma in different settings [8–12], these insights have not yet been systematically integrated into stigma reduction strategies. As Botswana faces a pressing need to improve adherence to HIV care among mothers in the postpartum period, it presents an ideal setting for a culturally tailored, stigma reduction intervention for women while they are actively engaged with health services during the antenatal period. Botswana has among the highest HIV prevalence worldwide with 24.3% of 15–49-year-olds infected [13], including 24% of pregnant women [14].

**Fig. 1:**
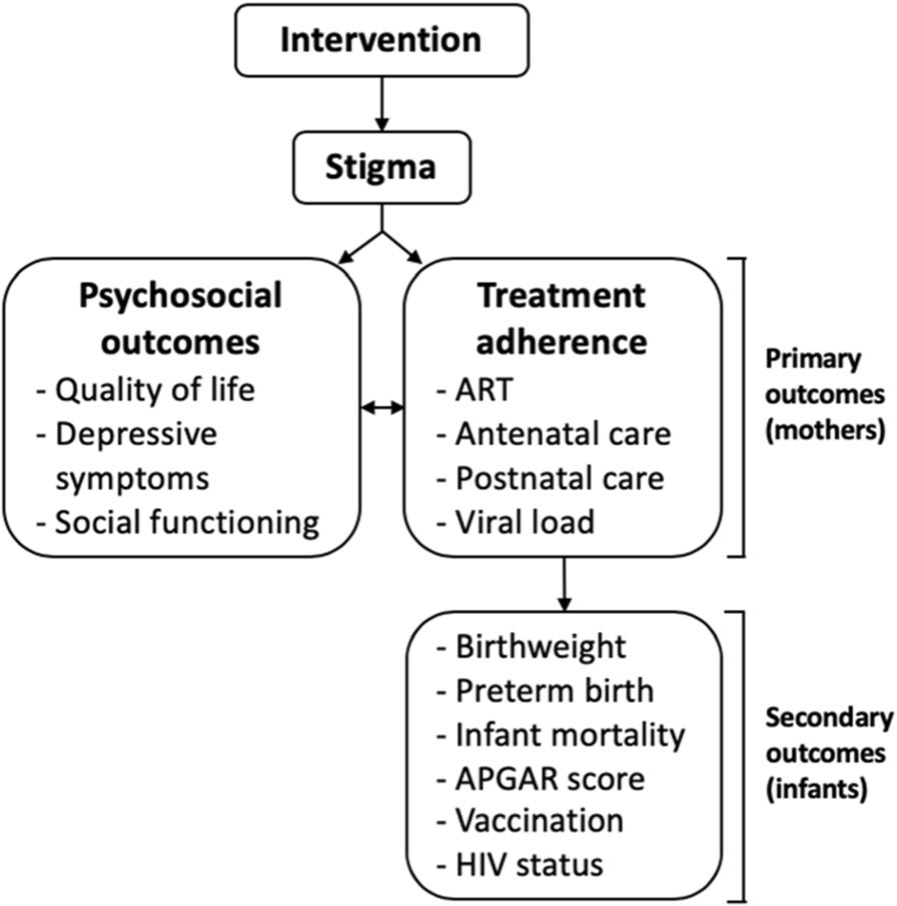
Model for stigma’s effect on treatment adherence

Since 2002, Botswana has provided a transformative national program to offer free antiretroviral therapy (ART) to all its citizens [15]. While eligibility was initially based on CD4 count, since 2016, Botswana has implemented a “Treat All” strategy to offer free ART immediately upon diagnosis of HIV [16]. Botswana is also one of the few sub-Saharan countries to offer free antenatal care for all mothers [17], with > 94% of mothers using antenatal services [14]. Prevention of mother-to-child transmission (PMTCT) of HIV is also a well-established and effective aspect of antenatal care in Botswana [14], where treatment follows the WHO “Option B+” guidelines of lifelong ART use for mothers regardless of CD4 count. Despite the availability of ART, particularly for mothers, challenges exist in maintaining postpartum adherence to ART in Botswana. Postpartum loss-to-follow-up and reduced ART adherence has been identified as a high priority worldwide [18]; a meta-analysis of 51 studies showed that ART adherence declined across countries from 76% prepartum to 53% postpartum [19]. While > 90% of pregnant mothers initiate ART in Botswana [14], results from a recent randomized controlled trial (RCT) suggest a significant drop-off in postpartum adherence [20]. Studies from neighboring South Africa, which like Botswana is a middle-income country that provides free ART [15], also show an increased loss-to-follow-up postpartum [21–23]. Explanations include mothers’ decreased motivation after having protected their neonate from HIV during pregnancy and birth and severe stigma leading to lack of disclosure of HIV [22, 24]. The traditional practice of Botsetsi [25], the semi-compulsory confinement where mother and infant do not leave the home up to 3 months postpartum, may also impact postpartum ART adherence in Botswana. Stigma’s effect on treatment adherence is represented in Fig. 1.

Growing evidence also suggests outcomes in HIV-exposed infants are poorer than unexposed infants [26–29]. The reasons for this are multifactorial and may include poorer birth outcomes (e.g., higher risk of preterm delivery, lower birth weight) [30, 31]. Decreasing stigma and increasing adherence to ART and antenatal care among pregnant mothers diagnosed with HIV, in addition to blocking mother-to-child transmission (MTCT), could improve birth outcomes for the infants (Fig. 1). We consider ART adherence and antenatal effects jointly because their provision is often linked in Botswana. Despite equivocal effects of ART adherence on birthweight [32], reducing stigma to improve mothers’ antenatal adherence to the WHO standard (≥ 4 antenatal visits, with the 1st visit in the 1st trimester [33–36]) could improve infants’ birth outcomes [36, 37]. Achieving stigma reduction at this crucial period is vital for future evaluation of longer-term infant outcomes when stigma may have greater adverse impacts (e.g., breastfeeding and MTCT). We hypothesize that by delivering “Mothers Moving towards Empowerment” (MME) during pregnancy, stigma reduction will persist postpartum and could help sustain ART adherence during a time period in which mothers remain especially vulnerable to stigma.

In addition to factors such as low education, poverty, and unmarried status [38], studies have identified cultural aspects of stigma related to gender that powerfully shape ART adherence. One meta-synthesis of 42 qualitative studies [38] in sub-Saharan Africa reported that, because HIV status is strongly linked with promiscuity, ART adherence elicits fear of stigma [39–46]. Further, stigma is linked with fear of losing romantic partners [47–50] and may lead to abandonment due to perceptions of infidelity [45, 51]. Women identified as having HIV also risk being blamed for contracting HIV and spreading it to their partners [47, 52, 53], due to perceptions that they were promiscuous and accountable for any wrongdoing in the family [52, 54, 55]. To further identify and target culturally salient aspects of stigma for women in Botswana, we utilized an influential theory, “what matters most” (WMM), which states that stigma is experienced most acutely when individuals are unable to achieve “full personhood” because they are unable to participate in activities that “matter most” in their cultural group. Restated, this theory indicates that stigma is felt most powerfully when it threatens an individual’s ability to take part in these daily activities thereby putting their “full personhood” as a valued member of their local cultural group at stake. Further, the WMM theory also helps identify the everyday cultural capabilities that *protect against* stigma by considering how achieving or maintaining the activities that are core to “full personhood” may enable an individual to resist HIV stigma [56, 57]. WMM therefore allows for the assessment regarding how cultural conceptions of “what matters most” within one’s cultural group directly interact with stigma [58] to exacerbate or mitigate it. Using this theory, our formative qualitative study found that achieving full status as a woman in Botswana is expressed through being a mother. Yet to properly care for children, many women in Botswana also depend on a male partner to provide financial support [59]. Thus, stigma may threaten personhood for many women in that being identified as having HIV may lead to abandonment by a male partner due to negative stereotypes, threatening women’s ability to care for children properly. On the other hand, a woman with HIV may be able to resist stigma if she is still able to fulfill the capabilities of being a “good mother,” if she so chooses, by bearing and raising children in ways that align with cultural expectations.

These culturally based dynamics can be further elucidated by examining their intersection with structural conditions such as the provision of healthcare [57]. Botswana is one of few sub-Saharan Africa countries to provide free antenatal care to all women, including those living with HIV. An unintended consequence is women also face greater vulnerability to stigma because PMTCT requires routine testing for HIV during pregnancy, thus leading to HIV status often being diagnosed among women first at time of pregnancy. We observed that because these women are often temporally identified as having HIV before their male partners, they are erroneously seen as having introduced HIV into their relationship [60]. The WMM approach enables reduction of stigma at this point of vulnerability by orienting an intervention towards resisting stigma that threatens women’s standing as a “good woman” in the community, thus addressing a core barrier to treatment adherence.

Stigma interventions have shown promise in reducing stigma and improving psychosocial outcomes [28, 29]. In particular, a key feature for mental illness stigma reduction is to use a “contact-based” modality via a peer co-leader who shares the stigmatized status but who has coped successfully with the stigmatized status. Contact reduces stigma by providing interaction with a person who moderately disconfirms preexisting stereotypes [30]. Per reviews of HIV stigma interventions [31–34, 61–65], only four studies described interventions that used contact with a peer co-leader with HIV to reduce stigma among PLWHA. Two were conducted in the USA and one in Vietnam [66], and all four showed improvements in stigma and psychosocial outcomes (Fig. 1). This contact-based strategy has only recently been introduced to sub-Saharan Africa [35] and has yet to be systematically evaluated. Lastly, no intervention has targeted stigma to improve postpartum ART adherence per a 2019 review of interventions for ART adherence among women [67].

This pilot study of MME collaborates with health clinics offering antenatal services under the Greater Gaborone District Health Management Team (GGDHMT) to conduct a pragmatic clinical trial [68, 69] of an innovative, culturally tailored stigma reduction intervention among pregnant mothers diagnosed with HIV. The intervention builds on prior evidence-based strategies while also integrating WMM for pregnant mothers in Botswana (see “Intervention rationale and components”). We named our intervention “MME” (pronounced “mm-eh”), short for “Mothers Moving towards Empowerment.” *Mme* is also a Setswana term used to address a respected woman. We utilize a pragmatic study design that retains all core elements except for randomization, which was not feasible among this complex population where we must group together women who are at approximately the same stage of pregnancy at the same time. Instead, we use systematic assignment by the experimenter to MME or a control group receiving treatment as usual (TAU). We measure primary and secondary outcomes at three time points (pre-intervention, immediately post-intervention, and at 16-week follow-up postpartum). All details regarding the protocol sections can be located through the SPIRIT checklist (Additional file 1).

## Methods/design

### Study overview

This study aims to test the MME intervention against TAU among pregnant mothers diagnosed with HIV and their infants, assessing outcomes during pregnancy and 16 weeks postpartum. We aim to reduce stigma, improve psychosocial outcomes, and maintain ART adherence among pregnant and postpartum women living with HIV who are especially vulnerable to HIV stigma. While we focus on maintaining ART adherence postpartum, we also explore the possibility of beneficial health effects on the newborn who could potentially benefit from stigma reduction and ART adherence in outcomes such as birth weight, preterm delivery, and timely achievement of developmental milestones.

### Ethics

This study was approved by the University of Botswana IRB (REF: UBR/RES/IRB/BIO/093, approved 2018-10-05), the Ministry of Health and Wellness’ Health Research and Development Committee of Botswana (HRDC) IRB (REF: HPDME:13/18/1, approved 2018-10-15), the Princess Marina Hospital IRB (REF: 5/79(456-1-2018), approved 2018-11-13), University of Pennsylvania IRB (REF: 829913, approved 2018-06-28), New York University IRB (REF: IRB-FY2018-1967, approved 2018-07-30), and the Greater Gaborone District Health Management Team (GG-DHMT, approved 2018-10-23). In addition, this study is funded by the National Institutes of Health/ Fogarty (R21TW011084). This study is registered with www.clinicaltrials.gov (NCT03698981). Finally, this study protocol has been reported in accordance with the Standard Protocol Items: Recommendations for Clinical Interventional Trials (SPIRIT) guidelines (Additional file 1).

### Setting

Gaborone is the largest city and capital of Botswana. The catchment area it services has a total population of approximately 270,000 [70, 71], and approximately 2800 Gaborone women give birth each year [72]. Approximately 30% of these women had a positive HIV diagnosis, with about a third already knowing their diagnosis and two thirds testing positive during the current pregnancy (2007–2008 data) [73]. HIV diagnoses for pregnant mothers are often first made through routine PMTCT at sexual reproduction clinics. Newly diagnosed pregnant women are referred to obstetric care at Ministry of Health (MOH) antenatal clinics and often receive HIV care at the Infectious Disease Care Clinic (IDCC) of Princess Marina Hospital for continued ART (including postpartum).

Recruitment takes place at a sub-group of government health clinics offering antenatal services in the Greater Gaborone District Health Management Team (GGDHMT) area and identified by GGDHMT as high-volume facilities. We cluster these clinics geographically such that each cluster (a) typically encounters patients meeting the eligibility criteria at a rate that allows for the formation of a group of 10–12 participants who could be allocated to receive the entire eight-session intervention prior to delivery (anticipated from the 36th week of pregnancy) and (b) has clinics near enough to one another such that participants recruited from any clinic in a cluster would not need to travel far to attend a cluster-wide intervention group session. This allows for the creation of two clusters: Tlokweng (3 clinics) and Gaborone-West (3 clinics).

The following recruitment procedures and eligibility requirements for participants were refined based on feasibility and finalized during piloting of the first intervention group.

### Recruitment

With support from clinical investigators, a Community Coordinator (CC) introduces the study to clinic staff at prospective recruitment sites and obtains permission to enroll from the facility head prior to beginning recruitment. The study asks clinic staff to approach all eligible women living with HIV registered for antenatal care, provide them with study information, and obtain their assent to be contacted by study staff.

Study staff visit the clinics weekly to obtain potential participant lists and contact them to confirm their eligibility, explain the purpose and procedures, and subsequently obtain informed consent for all study activities. Specifically, the CC meets with antenatal care (ANC) staff to arrange access to the ANC Register and to support identification of pregnant women meeting the eligibility criteria. Upon identification of potentially eligible women, the CC arranges for the health care worker to obtain assent to contact the mother directly. The CC begins direct engagement by reconfirming eligibility criteria are met—including the date of positive HIV test—prior to acquiring consent. With consent obtained, the study staff then completes the baseline assessment and discloses allocation to MME or TAU to the participant. Since this study focuses on the testing of intervention efficacy in a new context, we focus on recruiting into the MME group, and participants are placed into the control group based on overflow and availability (3:1). This also helps promote participant retention in the study.

Once the CC has identified a sufficient number of MME-allocated subjects within a cluster, study staff poll those participants to determine the week day suitable for the largest number of participants allocated to MME; the CC then makes a follow-up call to inform MME-assigned participants of the location, starting date, and anticipated end date of the group sessions. Sessions are scheduled to run at least once per week and may be scheduled twice per week if participants prefer, in order to maximize the possibility that all attendees benefit from all sessions of the intervention prior to delivery.

Participants are reimbursed for transportation costs for MME sessions and assessments at baseline, post-MME (intervention group only), and 16-week postpartum (approximately week 56 after calculated date of conception). Since MME is an optional stigma reduction to complement on-going clinical care, it does not entail ancillary or post-trial care.

### Eligibility

All pregnant women registering to receive antenatal care at these health clinics who are identified as having HIV and meet the inclusion criteria are eligible to be included in the trial; both primiparous and multiparous women may participate. In addition to being a pregnant woman receiving antenatal care and having HIV, participants are screened at the time of recruitment to ensure they meet the study inclusion criteria of being (a) 18–45 years of age, (b) a Botswana citizen, (c) English or Setswana speaking, and (d) no more than 28 weeks pregnant at the time of recruitment (to allow sufficient time to participate in the intervention prior to delivery). Women are expected to be in HIV care per national HIV treatment guidelines [16]; however, they are not required to be on ART treatment at the time of enrollment. The study excludes from participation (1) women who experienced a miscarriage prior to 28 weeks and (2) women unavailable to attend weekly 90-min sessions for eight sessions prior to week 36.

### Condition assignment and blinding

**Fig. 2:**
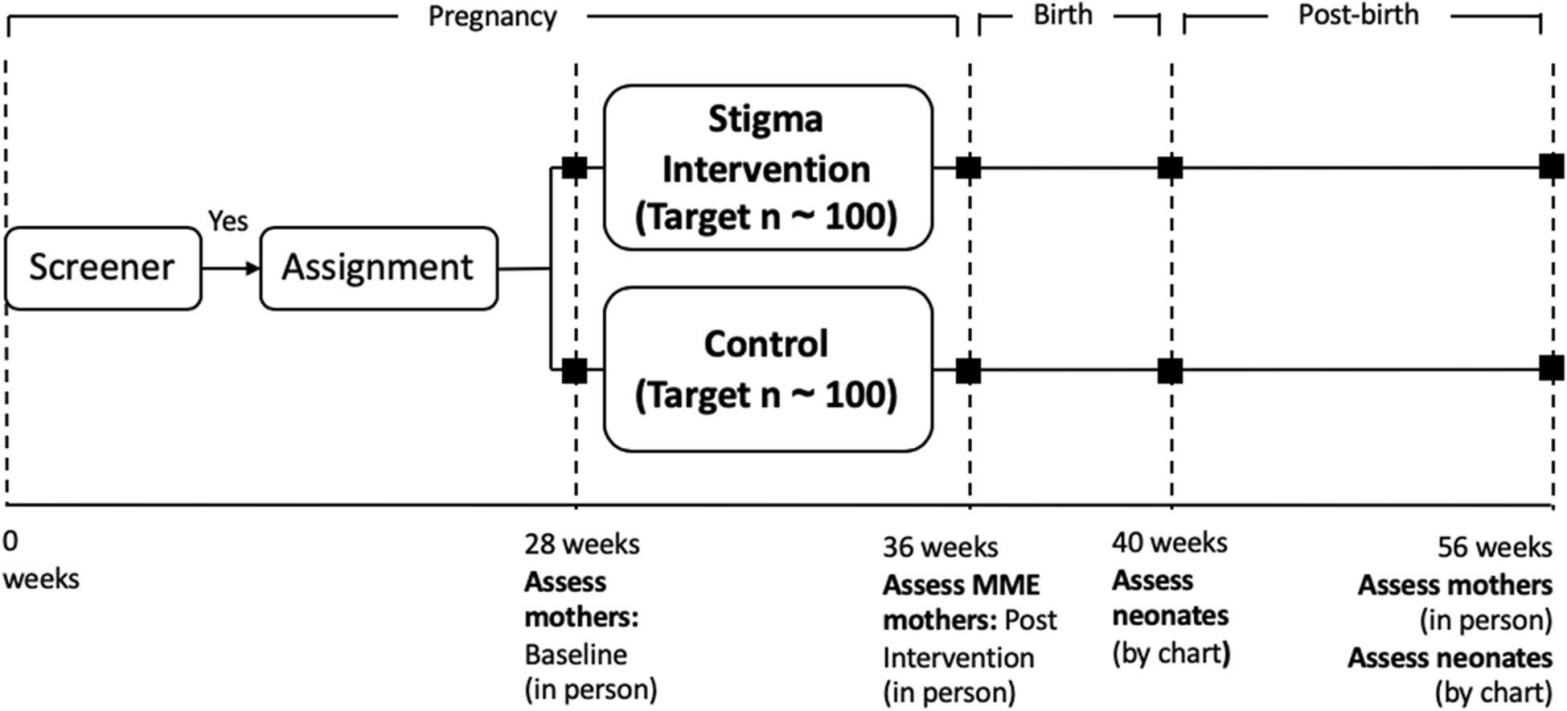
Schedule of enrollment, interventions, and assessments

Due to pragmatic challenges of recruiting pregnant women during the first 28 weeks of pregnancy, we are not able to use randomization. Participants who meet eligibility criteria are assigned by the research team to receive MME or TAU based on timing of recruitment (Fig. 2). Since we are not able to standardize the start of the intervention to the same week of pregnancy for participants, women are enrolled as long as it is feasible for them to complete eight sessions prior to week 36 of their pregnancy. In terms of blinding, participants and study staff administering the intervention are aware of treatment allocation; lead and senior investigators are blinded to treatment allocation. The biostatistician is not involved in the intervention assignment and is unblinded at the phase of analysis because the intervention group has measures immediately post-intervention that are not administered to the control group. Additionally, one of the primary analyses is to compare outcomes in the intervention group vs. the control group.

### Intervention rationale and components

The MME intervention integrates evidence-based stigma reduction techniques via three main components: psychoeducation for HIV [74–76], challenging inaccurate stereotypes of HIV [77, 78], and identifying behavioral coping responses for HIV-related discrimination [74, 77–81] which all have strong empirical support in improving one or more primary outcomes in PLWHA. In addition, each has been shown to reduce stigma [75–81], improve multiple psychosocial outcomes [75, 77, 78, 80, 81], and/or increase knowledge about ART treatment [76] for PLWHA. While each component shows individual effects, MME combined these components into a single, coherent stigma reduction package. Following work with other groups [82], we also culturally tailored the stigma reduction intervention to provide an option for participants to address “what matters most” for women in Botswana (i.e., fulfilling capabilities central to being “a good mother”). By discussing stigma and stereotypes related to HIV and incorporating what matters most to Batswana women, the intervention supports participants to develop behavioral coping responses to counter culturally salient stigmatizing cognitions and to restore status as being a “good mother” from the perspective of the respondent and group members. The major emphasis of the intervention focuses on these three aspects and targets resisting internalized stigma and discrimination related to HIV, ultimately restoring personhood to the participants. The intervention is designed to be led by co-leaders consisting of a mental health clinician and a peer with the same stigmatized status.

Two Principal Investigators (LHY, MB), both of whom are clinical psychologists, cognitive behavioral therapy (CBT) expert consultant (VJ), co-investigator psychiatrist (PO), HIV care pediatrician (TA), and epidemiologist (AH) (latter three on site in Botswana) comprised the Steering Committee guiding intervention design and adaptation. This committee held weekly Skype calls and met in person in Gaborone to adapt the MME manual based on a prior, evidence-based stigma reduction intervention [Yang LH, et al.: A culturally-based intervention to reduce stigma for Chinese immigrants with severe mental disorders: pilot evaluation and initial findings, submitted.] and incorporated the formative qualitative findings identifying “what matters most” (WMM) in this context. The 8-session MME curriculum utilizes the WMM approach to focus on stigma targets for overcoming obstacles to treatment adherence for pregnant mothers living with HIV, and whose effects are proposed to endure after delivery. MME integrates three components: (1) psychoeducation regarding causes, transmission, and treatment of HIV, specifically how adherence to ART and antenatal care acts to promote health for women living with HIV and PMTCT [83, 84]. We frame ART adherence spanning into the postpartum period as a maternal duty to raise a healthy child; (2) challenging stereotypes of women identified with HIV, such as promiscuity, that threaten core aspects of being a “good woman” and hinder treatment adherence; and (3) coping skills for HIV-related discrimination, including potential rejection or abandonment by male partners leading to discontinuing treatment adherence and impacts on raising children. The manual also discusses when it is adaptive to keep secret one’s HIV status, while also carefully identifying opportunities to disclose to others who are deemed safe, to increase social support for ART adherence.

In addition, we incorporated a section on general strategies for HIV status disclosure, emphasizing empowerment skills [85] when a mother wishes to disclose her HIV status. Should disclosure to a partner lead to domestic violence, the MME intervention provides all participants with contact details for a women’s shelter that provides services to women who have experienced domestic violence, as well as for psychological services at Princess Marina Hospital’s Psychiatric Clinic and the University of Botswana’s Psychology Clinic. Both intervention and control participants who disclose abuse to study personnel are referred for counseling via our expert in-country psychiatrist consultant (PO), or a qualified mental health practitioner he designates, for further counseling and linkage to available support services.

### Intervention procedure

The 8 sessions of the intervention occur weekly for about 90 min per session. Each session focuses on a particular theme including Introduction and Defining Stigma, Common Myths and Facts about HIV Transmission, Common Myths and Facts about Prevention of Mother-To-Child Transmission (PMTCT), The Road to Self-Acceptance and Freedom: Coping Strategies, Coping with Discrimination: Social Support and Self-Disclosure, The Road to Self-Acceptance and Freedom Part 2: Automatic Thoughts, What Matters Most, Review, and Graduation. Homework is assigned at each session and reviewed the next session. Certificates are issued to participants who complete the intervention (≥ 5 sessions) in a graduation ceremony. In consultation with local experts, we developed a concluding ceremony intended to convey “WMM” (i.e. “good mother” status) via the bestowing of ceremonial shawls that are typically provided when women become married and are ready to bear children. Finally, participants can leave this study at any time and, if choosing to withdraw, may decide to inform the study staff as to why, but are not required to do so.

### Training process

Following initial manual development, Steering Committee members convened in Botswana and trained a local clinical expert (SR), prospective psychology graduates trained in counseling, and peer co-leaders (i.e., women who had been identified with HIV during their own pregnancies) over an intensive 3-day period. The training included using the manual and stigma intervention skills in order to prepare the clinical and peer co-leaders to implement the intervention. To further refine the manual, co-leaders suggested modifications after each session, and extensive adaptations were made to further reflect cultural norms and concerns that were key to psychoeducation for HIV and stigma. After training, we expected ≥ 2 peers to agree to co-lead intervention groups. Preference was given to those who showed capacities to challenge stigma and who were willing to co-lead an intervention after individual stigma training. We envisioned a hierarchy developing in terms of capacity building in that the local clinical expert would then train a new cohort of peer co-leaders, with the clinical psychologist committee member (VJ) observing this process. The goal was to establish a “train the trainer” model in which there will be a cohort of local trainers on the MME manual to sustain this work and continue to reach women.

### Intervention fidelity

During the development phase of the MME manual, the Steering Committee included a licensed clinical psychologist consultant (VJ) who is a stigma and culture expert and specializes in cognitive behavioral therapy (CBT). This consultant was also on site during the co-leader training and interfaced directly with the peers, acting as the central evaluator for this pragmatic clinical trial. Her role also included developing the fidelity checklist for the MME manual. She monitors fidelity through weekly supervision calls with the local clinical expert and co-leaders, in which she provides a clinical lens to ensure the tenets of CBT are being met, confirm adherence to the MME manual per respective weekly sessions, and assess co-leader burnout. In regard to co-leader assessment, she additionally inquires about length of intervention sessions, quantity of sessions per week, and how peers are processing difficult information covered during the sessions.

### Comparator: treatment as usual

Control participants receive TAU, including using free ART and antenatal services as they wish. Budget constraints do not allow us to implement a time and attention placebo control condition; this threat to validity in this pilot study will be accounted for in a future RCT. Control condition participants are assessed on all primary outcomes at the same time points as the intervention group (Fig. 2), except for immediately following intervention.

### Dissemination policy

We will submit manuscripts to peer-reviewed journals with the Botswana team as lead and senior authors. We will also submit an NIH R01 proposing a multisite RCT of our stigma reduction intervention assuming success of this pilot study. In addition, we will communicate findings to participants as well as community and local stakeholders. At the end of the observation period, we will request consent from the participants to permit us to contact them using the contact information they have shared in order to invite them to a meeting where results will be communicated and feedback gathered. For the communities and local stakeholders, we have received support from the Botswana National Aids Coordination Agency (NACA) and we have invited them to participate throughout the study, including the design, intervention training, and results dissemination workshop. Once the study has been analyzed and findings are ready for sharing, we will approach them and the Botswana PEPFAR Coordination unit for opportunities to present the findings to key stakeholders (e.g. PEPFAR Implementing Partners). Finally, our team will also be submitting abstracts for both USA-based and international conferences to further share our findings.

Topics suggested for presentation or publication will first be presented to the Principal Investigator (LHY). Per suggested topic, explanation and justification for proposed authors will be submitted and reviewed by the full research team. Once a topic is approved, a writing committee will be established and the person who leads the presentation or publication may be considered as the lead author, but will need to confirm any final versions with the full team prior to submission. The Principal Investigator will be the lead author for the main outcomes study. All study staff who contribute intellectual elements to study design, study administration, data analysis, and manuscript development will be named as an author on all forthcoming publications.

The protocol team will make a de-identified data set publicly available a year after study completion in order to allow sufficient time for preliminary analyses and publications to be initiated by the study team. Contact with the Principal Investigator (LHY) will enable access to the dataset when available as well as data collection materials inclusive of informed consent forms.

### Assessments and outcome measures

All assessments are enumerated in Table 1, including their domain (name of the outcome), specific measurement, metric, method of aggregation, and time points. The three main time points are (1) baseline (< 28 weeks gestational age), post-intervention (immediately following completion of 8-session intervention, intervention group only), and follow-up (16 weeks postpartum). Primary outcomes for the mother include HIV stigma, psychosocial outcomes, treatment adherence, and HIV outcomes (e.g., viral load). Secondary outcomes for the infant include birth outcomes and early infant health. Stigma and psychosocial outcomes are collected via self-report questionnaires; all other outcomes are collected via health/medical records for the mother and infant. In addition, there are no additional studies using participants’ data and there are also no biological specimens that will be collected. If there are other studies to be conducted, there will be a separate procedure for additional consent.

**Table 1:** Overview of measures

Assessment measure	Baseline (< 28 weeks gestational age)	Post-MME	Follow-up (16 weeks postpartum)	Comparison metric
**Stigma outcomes**				
Berger HIV Stigma Scale	ALL	MME	ALL	1,2,3,4,5
WMM HIV Cultural Stigma Scale in Botswana (HCSS-B)	ALL	MME	ALL	1,2,3,4,5
Botho (3 items)	ALL	MME	ALL	1,2,3,4,5
**Psychosocial outcomes**				
Quality of life (PROMIS-8)	ALL	MME	ALL	1,2,3,4,5
Depressive symptoms (CES-D)	ALL	MME	ALL	1,2,3,4,5
Post-traumatic stress symptoms (PCL-5)	ALL	MME	ALL	1,2,3,4,5
Social functioning (WHODAS-12)	ALL	MME	ALL	1,2,3,4,5
Social support (6 items)	ALL	MME	ALL	1,2,3,4,5
**Treatment adherence**				
Antenatal adherence	ALL	MME	ALL	1,2,3,4,5
ART adherence (ACTG questionnaire)	ALL	MME	ALL	1,2,3,4,5
**Covariates**				
Demographic measures	ALL	MME	ALL	N/A
Alcohol use (AUDIT)	ALL	MME	ALL	N/A
Sexual behavior (5 items)	ALL	MME	ALL	N/A
Intimate partner violence (WAST)	ALL	MME	ALL	N/A
**Qualitative** (open-ended; differs at each time points)	MME	MME	MME	N/A
**HIV metrics:** CD4 count and viral load (IDCC records/electronic medical record system)			ALL	1
**Birth outcomes:** birth weight, gestational age, APGAR scores (Delivery Summary Sheet)			ALL	1
**Infant health:** infant mortality, infant HIV status, feeding method, vaccinations, hospitalizations (dates, diagnosis) (“Under 5 Card”: asked from mother at 4 month follow-up)			ALL	1

1 Bivariate comparison of mean (median) scores at 16-week follow-up between intervention and control group

2 Bivariate comparison of change in mean (median) scores from baseline to 16-week follow-up between intervention and control groups

3 Bivariate comparison of mean (median) scores at baseline and post-MME within intervention group

4 Bivariate comparison of mean (median) scores at baseline and 16-week follow-up within intervention group

5 Bivariate comparison of mean (median) scores at post-MME and 16-week follow-up within intervention group

*ALL* all participants

*MME* only intervention participants

### Primary outcomes

Primary outcomes among mothers include (i) reducing self-stigma, (ii) psychosocial outcomes (e.g., quality of life, social functioning), and (iii) treatment adherence (antenatal care, ART), which should result in improvements in HIV outcomes (CD4 count, viral load).

#### Stigma

Stigma is measured by two validated scales and three additional items. The Berger HIV Stigma Scale is an established 40-item scale to measure stigma among PLWHA that has good reliability (alpha = 0.96) and validity [86]. The WMM HIV Cultural Stigma Scale in Botswana (HCSS-B) is a new 20-item scale developed specifically to measure culturally salient and protective aspects of stigma for HIV-positive women in Botswana, which has demonstrated good reliability for each of two subscales (alpha = 0.90 each) and validity [87]. Three specific study-developed items gauge how stigma interacts with Botho, a Setswana concept related to the qualities of being a good person (e.g., Botho phrase—“A person is a person because of other people”—*motho ke motho ka batho*).

#### Psychosocial outcomes

Five distinct psychosocial outcomes are measured: (1) Quality of Life via the Patient-Reported Outcomes Measurement Information System (PROMIS) 8-item Ability to Participate in Social Roles and Activities Short Form, designed and validated for use among general populations and individuals with chronic conditions [88, 89]; (2) Depressive Symptoms via the widely used 20-item Center for Epidemiological Studies Depression Scale (CES-D), validated in both general and psychiatric populations [90]; (3) Post-Traumatic Stress Symptoms via an abridged, 8-item version of the Post-Traumatic Checklist for DSM-5 (PCL-5). The full 20-item version has been validated for provisional diagnoses in military and non-military populations [91]. (4) Social Functioning via the 12-item World Health Organization Disability Assessment Schedule (WHODAS) 2.0, designed to be used across all diseases and populations and previously validated in multi-country clinical trials [92]; and (5) Social Support via a 6-item measure of perceived availability of social support that asks participants their confidence that, if needed, adequate support would be available to them across six different domains [93].

#### Treatment adherence

Antenatal adherence is measured by tracking the number of antenatal visits to the clinic, both objectively using the “maternal clinic card” that is filled out by doctors and that mothers carry, and subjectively from participant self-report. ART adherence is measured using the AIDS Clinical Trial Group (ACTG) Adherence Follow-Up Questionnaire, which asks participants details about their HIV medication use patterns over the last 4 days and any reasons for non-adherence [94].

#### HIV metrics

The two most common biological measures of HIV are CD4 count and viral load, which are both extracted from the IDCC electronic medical record system along with the date(s) of each test and the date of ART initiation. Due to limited resources, we are not able to measure these metrics directly and therefore rely on clinical measurement and documentation (noting that documentation for viral load may be sparse due to inconsistent following of recommendations of a viral load test in the 3rd trimester). When data are available, the dates may not align with any of our time points. Due to these pragmatic concerns, we opt to collect these metrics only once at the end of the study and then ascertain how best to proceed for analysis.

### Secondary outcomes

Because increasing adherence to antenatal care and to ART is hypothesized to improve birth outcomes such as low birthweight and preterm delivery [31] (Fig. 1) and early infant health such as survival and HIV status, we examine these in exploratory analyses (study not powered for these outcomes).

#### Birth outcomes

All birth outcomes will be extracted from the Delivery Summary Sheet, including the infant’s date of birth, birth weight (grams), gestational age (number of weeks), date of discharge (or death, if child died before discharge), and APGAR scores at 1, 5, and 10 min [95].

#### Infant health

All infant health metrics are extracted from the “Under 5 Card,” which is carried by the mother and filled out by a doctor to track infant’s developmental outcomes. We chose the 16-week time point for follow-up because that is the first time that all measures are recorded on this card. This includes infant mortality (< 16 weeks), infant HIV status (i.e., if mother-to-child transmission occurred), feeding method (breastfeeding, formula, both), vaccination schedule, and hospitalization for any illnesses (up to three diagnoses and admission/discharge dates).

### Covariates

Additional measures include sociodemographic information (date of birth and age; racial, ethnic, and/or tribal background; sex at birth and gender identity; sexual orientation; relationship status; highest education level; estimated total family income; employment pattern; money for basic necessities; assistance from government or local community leadership; membership in private medical aid; homelessness; and housing status, including household items). Three additional scales are included: (1) alcohol use via the 10-item Alcohol Use Disorder Identification Test (AUDIT) [96, 97]; (2) sexual behavior via five questions about number of sex partners in the past month; how often used condoms during sex with regular partner(s) in past month; how often used condoms with casual partners in past month; how often used condoms for paid sex in past month; and how often anal sex in past month; and (3) intimate partner violence via the 8-item Woman Abuse Screening Tool (WAST) [98]. These covariates will be collected at baseline and will be used in a purely descriptive fashion to characterize the sample.

### Intervention process measures

For participants of the MME intervention, we use a fidelity checklist to monitor that the core components of each session are implemented and track any deviations. This is confirmed during weekly supervision meetings. We also track attendance to ascertain the dosage of MME for each participant. At baseline, we qualitatively ask participants what they hope to gain from the intervention, and at completion, we ask them both what they gained and any changes they would recommend for the future. Because one of the primary goals of the intervention is building social support, at 16-week follow-up we also qualitatively ask the participants if they have stayed in continued contact with their fellow group members, the group leaders, and/or any other counselors or support groups.

### Data management

As part of capacity building, the data center is located at the University of Botswana. All data is collected and entered by trained research staff to be stored virtually on the University of Pennsylvania (UPenn) instance of REDCap. All assessments are recorded on the final version of the case reporting forms (CRFs) and inputted into the secure web-based data management system operated by the Data Management Unit (DMU) of the University of Botswana.

During data collection, security of the web-based data entry system is maintained through the use of a double authentication system. Each system user is assigned two username/password combinations. The first username/password combination is one selected by the registered user and provides access to the main data management website. Access to the website is limited to authorized users by the following condition: (1) all users must become registered with the DMU and (2) access to the DMU website will be restricted to study personnel who work with the UPenn instance of REDCAP and must be approved by the Director of DMU. The second username and password are assigned by the DMU and provides access to the data entry portion of the website. Strict standards are enforced when providing access to the data entry system. A co-investigator validates users in writing for access and security assignment. Additionally, all users of the web data entry system are required to complete CITI training and certification. The data center is unblinded and will adhere to the Data Safety and Monitoring Plan (DSMP), which informs reports, database locking, and analysis. A Data Safety and Monitoring Board (DSMB) will meet every 6 months to monitor adverse events (AEs) and safety outcomes. The DSMB consists of 3 experienced, doctoral-level HIV researchers (> 5 years of research in the field) who will meet every 6 months to review any adverse events and safety outcomes, and independently determine whether study activities will (1) continue as planned or (2) stop pending full IRB review and only continue pending subsequent IRB approval. Study personnel will abide by the determination of the DSMB.

In regard to adverse events, we anticipate two events that may occur, intimate partner violence, and depression and suicidal ideation. In order to protect the privacy of participants and to mitigate the possibility of intimate partner violence, protections for participant privacy will include the following: contact only with the participant; no study-related information will be left on answering machines or other devices; no study-related information will be left with individuals who reside within the household. These procedures will also be followed when participants are contacted about the longitudinal evaluation at 16-week follow-up. When an instance of intimate partner violence is disclosed either during an anti-stigma group session or during the baseline or follow-up evaluation, the study team member who identified the event will report the date, description of the event, duration, severity, measures used to ameliorate effects of the event, and type of referral. The Project Director (AHO) will be notified immediately, and the report form will be reviewed and signed by the PIs (LHY and MB) within 24 h who will be responsible for ensuring that appropriate actions have been taken. The participant will be immediately referred to psychiatric counseling by a clinician under the supervision of our expert in-country psychiatrist Consultant (PO) and other supportive services (e.g., domestic violence support group). The clinician will conduct an in-person clinical evaluation within 24 h, and if the participant is found to be experiencing significant psychiatric distress, this clinician will immediately connect the participant with clinical services in the Princess Marina Hospital Department of Psychiatry, which will address any distress via in-person outpatient meetings and continuing therapy sessions as needed.

Study participants may disclose depression and suicidal ideation via the following methods: (1) during the anti-stigma intervention group sessions and (2) via the CES-D (see “primary outcomes”) measure at baseline and at 16-week follow-up (i.e., if any participant scores > 16 on the CES-D, the cut-off indicating probably clinical depression). If either of these conditions take place, then the procedures for intimate partner violence as described above will be initiated, including notifying the Project Director and PIs and the conducting of a clinical evaluation, within 24 h. If needed, the participant will be connected with a treating clinician in the Princess Marina Hospital Department of Psychiatry and provided with continuing therapy sessions as needed. The evaluating clinician will determine based upon his/her clinical evaluation whether the depression and/or suicidal ideation reaches a magnitude that necessitates removal from study participation. If removal from the study takes place, that participant will no longer attend stigma intervention sessions or participate in the follow-up evaluation; they will also be given the option to remove their data from the study. Regarding reporting of adverse events in trial publications, within the papers a separate section will restate how we report/record adverse events, a brief description of said events, and then how the study team addressed them.

Data analysis will be conducted by the biostatistician (MG). As part of an annual data audit, a clinical research compliance assessment will take place within 90 days of the IRB approval expiration date to assess the prior year’s activity. This is done by the UPenn in-country regulatory staff that are independent from the study team. This audit uses UPenn compliance tools that include a plan for reassessment if there are any issues that need to be addressed; the Research Director for the Botswana-UPenn Partnership will sign off on this process. The results are shared with the study team and all documentation will be completed by the Principal Investigator (LHY) and study staff. In addition, and in regard to data completeness and quality, we are conducting preliminary analyses in order to examine the data in real time and also utilize REDCap data completeness checks. The Principal Investigator (LHY) will also have access to these analyses. Should the trial need to be terminated, the Principal Investigator is responsible to make the final decision to terminate the trial.

All hard copy data are kept in locked filing cabinets in a locked office at all times. All data, including viral load and CD4 counts, are kept according to study participant number (de-identified). De-identified data including all paper interviews are kept separate from confidential data, such as contact information. Data is entered at University of Botswana situated offices and is subsequently stored at the Botswana-UPenn office. All data, once returned to the BUP study offices, are not be permitted to be removed. After data cleaning and analysis activities have concluded, all identifiable data, including patient locator forms, will be destroyed.

### Statistical analysis

We will examine baseline demographic characteristics of the intervention and control group and statistically test for differences between the groups on key characteristics to characterize the samples. Given the relative small sample size, nonparametric tests will be used to examine differences between groups. We will use bivariate analysis to compare primary outcomes of the mother’s reduced self-stigma, psychosocial outcomes, and treatment adherence, and secondary outcomes for infants including birthweight, timing of delivery, and developmental milestone between the treatment and control group using an intent-to-treat analysis at baseline and 4-month follow-up. Regarding missing data, we will conduct analyses based on complete data on each outcome and we will remove anyone if they do not have complete data for the outcome being assessed. Sensitivity analyses will be conducted based on the dose of intervention received using several different cut-points: (1) attended at least one session (yes/no), (2) number of sessions attended (count), (3) attended five or more sessions (yes/no), and (4) attended last session for graduation (yes/no). For bivariate tests, Wilcoxon rank-sum test will be used to examine differences in continuous variables between the intervention and control group. The chi-square test will be used for categorical data (Fisher’s exact test will be used when there are small cell counts). In addition, we will conduct exploratory analyses among intervention group participants in a pre/post-intervention analysis. Primary outcomes will be examined using the Wilcoxon signed-ranked test to examine difference among intervention participants from pre-intervention to post-intervention. Furthermore, we will examine whether there is correlation within groups and, if so, will then control for group membership in the analysis.

We will then build exploratory linear (continuous outcomes) or logistic (dichotomous outcomes) regression models to examine the effect of study arm on primary and secondary outcomes, and examine mediating effects of stigma on primary outcomes in mothers and exploratory outcomes in infants. We will prioritize objective measures in examining antenatal and ART outcomes and use self-report measures as a check on these findings. We will examine changes in predictors (e.g., stigma) between time points by creating difference variables and also examine these predictors as time-varying covariates in the regression model using generalized estimating equations (GEE) and structural equation models (SEM). A focus will be to examine whether changes in primary outcomes in mothers from baseline to post-intervention predict changes in primary outcomes at follow-up. If possible, SEM will also be used to examine structural relationships between latent constructs in the conceptual framework (e.g., among stigma subscales) and measured variables. Statistical significance will be assessed as *p* < 0.05. Statistical analysis will be conducted in Stata 16.

### Sample size and power

Power calculations were done using Power Analysis and Sample Size Software (PASS 2019) [99]. Given the exploratory and pragmatic nature of the study, we calculated power based on the Wilcoxon rank-sum (Mann-Whitney *U*) test for primary outcomes (measured continuously) based on an intent-to-treat analysis. We assume a 3:1 rate of intervention to control participants. Example group sample sizes of 5 (control) and 15 (intervention) achieve > 85% power to detect a median difference of 1.0 using a two-sided Mann-Whitney *U* or Wilcoxon rank-sum test assuming that the actual data distribution is normal when the significance level (alpha) of the test is 0.05 [100–104]. An example sample size of 40 achieves > 80% power to detect a Spearman rank correlation of >|0.45| using a two-sided hypothesis test with a significance level of 0.05. These results are based on 5000 Monte Carlo samples from the bivariate normal distribution under the alternative hypothesis [105, 106]. We will also calculate power post hoc based on the data collected in this study (actual sample size and effect size) before we begin analysis.

## Discussion

We take advantage of this rare opportunity to adapt, train, and evaluate a culturally based, HIV stigma reduction intervention to maintain ART adherence among those who are especially vulnerable—pregnant women with HIV. In the context of Botswana, a country with high HIV prevalence but also national programs to provide free ART and antenatal care [107], reducing stigma addresses a core barrier to postpartum ART adherence for women living with HIV. While increasing efforts have been made to learn how to reduce stigma and increase adherence for this vulnerable population, very few interventions have been shown to reduce HIV stigma into the postpartum period. Due to structural conditions, women face greater vulnerability to stigma because routine testing for HIV during antenatal care leads women to more frequently be identified as having HIV before their male partners and consequently be blamed for bringing HIV to the relationship. Based on our formative qualitative work to adapt the MME intervention for what matters most for women, we find that the desire to fulfill “being a good mother” may motivate mothers to remain ART-adherent when pregnant [24] but could wane postpartum and is threatened by these sources of stigma. We aim to extend positive effects of our culturally tailored stigma intervention into postpartum by both countering harmful stereotypes and informing about the benefits of continued ART postpartum. By doing so, we aim to reduce stigma, improve psychosocial outcomes, and maintain treatment adherence among women who have been identified as having HIV during pregnancy. We will also consider the potential intergenerational benefits for infants, not only in reducing mother-to-child transmission of HIV but for other birth and early health outcomes [108].

Potential limitations of this study start with challenges in recruitment and retention. From the outset, difficulties in recruiting only primiparous women led us to expand our criteria to multiparous women. However, we can imagine women who have previously given birth may differ from those who have not. The challenges of rolling recruitment of pregnant women during the narrow window of eligibility of the first 28 weeks have also prevented us from randomizing the treatment and control groups; however, we are still allocating participants to groups systematically and will adjust for any baseline differences in analyses. Pregnant women have many competing demands on their time and so it can be challenging to participate in an 8-session program. We have been responsive to requests from groups that are interested in having sessions twice weekly. It would also be helpful to know which aspects of the interventions were most effective; however, since the components are integrated together, we will not be able to isolate the effects to specific components. Given the formative work and adaptation to this specific context, whether the findings themselves are generalizable must be empirically tested; however, the approach and standardized methods we have developed can be used to replicate the process in new settings. Finally, the child outcomes are exploratory, but if promising, then future studies should be powered to detect clinically meaningful differences in these variables.

Currently, little is known about how to reduce HIV stigma that interferes with postpartum treatment in a context of free antenatal care and ART treatment. This study offers a model for how to use formative research and the WMM theoretical framework to identify culturally meaningful aspects of stigma and adapt evidence-based stigma reduction intervention components for women living with HIV in Botswana. While we realize that changing stigma in other stakeholders (e.g., family, community members, healthcare providers) is crucial, here we choose to alleviate stigma among pregnant women living with HIV whom we have identified as having particular vulnerabilities towards stigma, in part due to inadvertent effects of current HIV treatment policies. The intervention explicitly focuses on this by helping women resist stigma that threatens their moral standing in the community and emphasizes how continued ART during postpartum contributes to being a good mother. By training peer co-leaders, we build on existing resources and enhance local capacity. We envision a cohort of local trainers with the ability to sustain the work and continue to reach women after the study ends. Finally, we are conducting a rigorous evaluation of the intervention. To our knowledge, this is one of the first studies to systematically evaluate a stigma reduction intervention using a peer contact-based approach in sub-Saharan Africa [35], and has the potential to be one of the only stigma interventions to show stigma-reducing effects into the postpartum period. We use the most robust pragmatic study design that is feasible in terms of recruitment and implementation. Although we lack randomization, we include a control group, systematically allocate to intervention or control, blind all research staff except those administering the intervention, conduct assessments at multiple time points, include both subjective and objective measures of health and wellbeing, and consider potential intergenerational effects with both mother and infant outcomes. We also track process outcomes including fidelity and dosage. Together, this will help inform further refinement of the MME intervention and preparation for a large-scale, multisite, randomized controlled trial in the future.

### Trial status

As of submission, this protocol is version 2 and is registered with Clinical Trials, www.clinicaltrials.gov, (NCT03698981) as of October 8, 2018. Recruitment began in March 15, 2019, and will be completed as of October 31, 2020.

## Supplementary information

**Additional file 1.** SPIRIT 2013 Checklist

**Additional file 2.** Informed Consent Form

## Data Availability

Access to a de-identified dataset will be made available upon reasonable request following primary manuscript publication. The Principal Investigator (Lawrence Yang, PhD, Lawrence.yang@nyu.edu) can be contacted for details.

## Abbreviations

MME: “Mothers Moving towards Empowerment”
TAU: Treatment as usual
WMM: What matters most
ART: Antiretroviral therapy
HIV/AIDS: Human immunodeficiency virus infection/acquired immune deficiency syndrome
CD4 count: Cluster of differentiation 4
MTCT: Mother-to-child transmission
PMTCT: Prevention of mother-to-child transmission
PLWHA: Person living with HIV/AIDS
RCT: Randomized controlled trial
MOH: Ministry of Health
IDCC: Infectious Disease Care Clinic
GGDHMT: Greater Gaborone District Health Management Team
ANC: Antenatal care
DSMB: Data Safety and Monitoring Board
DMU: Data Management Unit
CRFs: Case reporting forms
CC: Community Coordinator
CBT: Cognitive behavioral therapy
BUP: Botswana Upenn Partnership (BUP)
APGAR Score: Appearance, Pulse, Grimace, Activity, and Respiration
PROMIS: Patient-Reported Outcomes Measurement Information System
WHODAS 2.0: World Health Organization Disability Assessment Schedule 2.0
QOL Scale: Quality of Life Scale
CES-D: Center for Epidemiological Studies Depression Scale
PCL-5: Post-Traumatic Checklist for DSM-5
ACTG: AIDS Clinical Trial Group
IPV–WAST: Intimate Partner Violence – Women Abuse Screening Tool
GEE: Generalized estimating equations
SEM: Structural equation models
PASS 2019: Power Analysis and Sample Size Software 2019
PI: Principal Investigator
