# Supplementary material for: ‘Mothers moving towards empowerment’ intervention to reduce stigma and improve treatment adherence in pregnant women living with HIV in Botswana: study protocol for a pragmatic clinical trial
Source: Trials. 2020 Oct 7;21:832. doi: 10.1186/s13063-020-04676-6 (PMC7542742; doi:10.1186/s13063-020-04676-6)
Supplement: Supplementary file 2 — Additional file 2. Informed Consent Form [file 13063_2020_4676_MOESM2_ESM.docx]

**INFORMED CONSENT FORM**

**RANDOMIZED CONTROLLED TRIAL TO REDUCE STIGMA AND IMPROVE TREATMENT ADHERENCE IN HIV+ PREGNANT WOMEN IN BOTSWANA**

You are invited to take part in a research study being conducted at Princess Marina Hospital and selected clinics of the Greater Gaborone Health District. This research is being done in collaboration with the University of Botswana, the University of Pennsylvania, and New York University. This form is to explain to you what we are going to do and why we need to ask for your consent.

**WHY IS THIS STUDY BEING DONE?**

The purpose of this study is to reduce HIV-stigma and improve anti-retroviral treatment (ART) compliance in Botswana. We are studying pregnant mothers' ART compliance after delivery because this is a time when women are more likely to stop ART. We also want to know how reducing HIV-stigma may improve infant health such as birth weight, preterm delivery and achieving age-appropriate milestones.

**WHY AM I BEING ASKED TO BE IN THIS STUDY?**

Because we are selecting pregnant women, aged 18 to 45, who have Botswana citizenship, speak either English or Setswana, and who are diagnosed as HIV+ and receiving care through IDCC.

**HOW MANY PEOPLE WILL BE ASKED TO BE IN THIS STUDY?**
A total of about 220 participants will be enrolled in Botswana.

**WHAT ARE THE ALTERNATIVES TO BEING IN THIS STUDY?**

You do not have to participate in this study and if you choose not to participate all the services you receive at the hospital will continue and nothing will change. You will still have your normal doctor’s appointment and care.

**WHAT WILL I BE ASKED TO DO IN THIS STUDY?**

We will be comparing a group of HIV+ pregnant women who complete a new type of education and counseling program (called the intervention group), to a group of women who complete standard treatment without the new program (standard treatment group). If you agree to participate in the study, research staff will randomly assign you to be part of the intervention group or the standard treatment group. This is like flipping a coin to choose one of two actions, which means you can choose to withdraw from the study at any time, but you will not be able to choose which group you are in. ***Intervention group procedures****:* Our 8-session group intervention will occur weekly for 60-70 minutes per session. Women will meet in groups of 9 to 10. A peer (an HIV+ mother) who has been trained will be helping the leading clinician to guide the group intervention sessions. Homework is assigned each week and reviewed the next session. Certificates will be awarded to participants who complete the intervention. ***Standard treatment group procedures:*** Participants in the standard treatment group will receive standard treatment, including using free ART and antenatal services as they wish.

Participants will be asked to fill out in-person questionnaires (asking for information on antenatal care compliance, stigma, quality of life, depressive symptoms, social functioning, social support, sexual behavior, and alcohol use). Questionnaires will be completed at a study site that is unaffiliated with Princess Marina Hospital or at your home, as preferred. If it is not possible to complete the questionnaire in person, a member of the study team may contact you to complete the questionnaire by telephone. Questionnaires will be completed at the following times-points: (1) before the start of the intervention; (2) at the completion of the intervention; (3) 4-weeks after the completion of the intervention; and (4) 20-weeks after the completion of the intervention. Participants in the standard treatment group will be evaluated at the same time-points. Other information collected (including HIV-medication adherence, CD4 count, and viral load) will be taken from your secured records in the integrated patient management system (IPMS). Information about your child (length of pregnancy, date of birth, birthweight, date of hospital discharge, APGAR score, and mortality) will be collected from the “Under 5 card.” Information about infant health will be collected 4-weeks and 20-weeks after the completion of the intervention, participants in the standard treatment group will be evaluated at the same time-points. Hospital records will provide infant data on length of pregnancy, birthweight, physical health, HIV status, vaccination status, and mortality. Information about method of delivery, delivery complications, maternal medical conditions, and breastfeeding, will also be collected. Please note that no identifiable health information will be shared with outside institutions.

**FOR HOW LONG SHOULD I EXPECT TO PARTICIPATE IN THIS STUDY?**

All consenting participants will be asked to complete questionnaires at 28 weeks of pregnancy and 16 weeks after the birth of your baby. Participants assigned to the Intervention Group will also meet weekly between weeks 28 and 36, and be asked to complete a questionnaire at the end of the intervention. Participants should expect to participate for about 28 weeks, however this period may be shorter depending on how long their pregnancy is.

**ARE THERE ANY RISKS TO ME AND MY CHILD?**

This study presents no more than low risk to you and your child. However, there are some limits to our ability to protect your confidentiality. These limitations include: (1)  because of the group-format of the intervention - there is a chance that participants might tell others about what happened in the intervention group (including sharing another person’s HIV+ status), and (2) disclosure of violence within the home is required to be reported to study staff who will conduct an in-person clinical evaluation with you and will either directly connect you to a treating clinician at Princess Marina Hospital or will refer you to psychiatric counseling and other services  such as domestic violence support groups.

To reduce the likelihood of breaching confidentiality, intervention groups will be consistently reminded that information shared during the group intervention is confidential and should not be shared with others. To further protect your confidentiality, we will abide by strict guidelines for maintaining confidentiality and securing data. Each research participant is assigned a unique study number that will be used to identify her data. All identifying information (name, address, telephone number) will be kept in a separate file apart from the research data. At no time will research data be linked with names, addresses, or telephone numbers, nor will any participants be identified in the presentation of results.

**ARE THERE ANY BENEFITS TO ME AND MY CHILD?**

There may be no direct benefit to you or your child by being in this study. The information from this study may help inform doctors in the future to promote better ART-compliance among this population.

**WILL I BE COMPENSATED FOR PARTICIPATING?**

Yes. You will be reimbursed for travel-costs related to participation in this study. No additional compensation will be provided.

**WILL THERE BE ANY COSTS TO MY CHILD OR ME?**

No.

**WILL INFORMATION FROM THIS STUDY BE KEPT CONFIDENTIAL**?

Yes. While the study team cannot guarantee total confidentiality, as information may be disclosed by other study participants, intervention groups will be consistently reminded that information shared during the group intervention is confidential and should not be shared with others. Strict guidelines for maintaining confidentiality and securing data are in place for this study. Each research participant is assigned a unique study number that will be used to identify her data. All identifying information (name, address, telephone number) will be kept in a separate file apart from the research data. At no time will research data be linked with names, addresses, or telephone numbers, nor will any participants be identified in the presentation of results.

**WHOM MAY I CONTACT FOR MORE INFORMATION?**

You can call any of the investigators listed here to tell him/her about a concern or complaint about this research study. The investigators can be reached as follows:

1. **Ari Ho-Foster**

Email: [ahofoster@fastmail.fm](mailto:ahofoster@fastmail.fm)

Telephone:  +267 355 4553 / +267 72515786

1. **Tadele Benti**

Email:   [tadele3300@yahoo.com](mailto:tadele3300@yahoo.com)

Telephone: +267 7 423 1505

If you want to talk with someone other than those working on the study, you may contact the Ministry of Health’s Health Research Development Committee (HRDC) with any question, concerns or complaints at: 391 4467. You may also contact Ms Julia Gaorekwe at the University of Botswana Office of Research and Development (ORD): 355 2900/2902.

**CAN I CHANGE MY MIND ABOUT PARTICIPATING?**

Yes. You may change your mind at any time without any effect on your care or that of your child. Investigators Ari Ho-Foster or Tadele Benti should be notified of your decision to withdrawal from the study (refer to the contact information listed above). This can be done either by phone or email.

**STATEMENT OF CONSENT**

The procedures, risks, and benefits of this study have been told to me and I agree to be in this study and sign this form. My questions have been answered. I may ask more questions whenever I want. I do not give up any of my child’s or my legal rights by signing this form. A copy of this signed consent form will be given to me.

___________________________________

Subject’s Name

___________________________________ ____________________________________

Subject’s Signature Date

_________________________________ ____________________________________

Signature of Interviewer Date

_________________________________ ____________________________________

Signature of Witness (if illiterate) Date
